# Impact of N-PEP-12 Supplementation on Attentional Performance and Mental Wellbeing in Healthy Adults with Subjective Cognitive Complaints: A Randomized, Placebo-Controlled Trial

**DOI:** 10.3390/nu18162739

**Published:** 2026-08-21

**Authors:** Diana Chira, Nicoleta Jemna, Olivia Verișezan-Roșu, Ana-Maria Buruiană, Ștefan Strilciuc, Vlad-Florin Chelaru, Simona Pârvu, Dafin Fior Mureșanu

**Affiliations:** 1 Department of Neurosciences, Iuliu Hațieganu University of Medicine and Pharmacy, Victor Babeș 43, 400012 Cluj-Napoca, Romania; diana.chira@brainscience.ro (D.C.); nicoleta.jemna@brainscience.ro (N.J.); vlad.chelaru@brainscience.ro (V.-F.C.); dafinm@ssnn.ro (D.F.M.); 2 RoNeuro Institute for Neurological Research and Diagnostic, Mircea Eliade 37, 400354 Cluj-Napoca, Romania; olivia.rosu@brainscience.ro (O.V.-R.); anamaria.buruiana@brainscience.ro (A.-M.B.); 3 Research Center for Functional Genomics, Biomedicine and Translational Medicine, Iuliu Hațieganu University of Medicine and Pharmacy, Gheorghe Marinescu 38, 400337 Cluj-Napoca, Romania; 4 Department of Translational Neurosciences, MEDFUTURE, Iuliu Hațieganu University of Medicine and Pharmacy, Gheorghe Marinescu 38, 400337 Cluj-Napoca, Romania; 5 Department of Complementary Sciences, Discipline of Hygiene and Medical Ecology, Carol Davila University of Medicine and Pharmacy, 8 Eroii Sanitari Boulevard, 050474 Bucharest, Romania

**Keywords:** subjective cognitive complaints, N-PEP-12, attention, memory, mental wellbeing

## Abstract

**Background**: Subjective cognitive complaints (SCCs) are common in middle-aged and older adults and may reflect early cognitive changes, alongside alterations in stress, mood, sleep, and quality of life. N-PEP-12 is a peptide-based nutritional supplement with potential neuroprotective effects, but evidence of its benefits in healthy adults with SCCs remains limited. **Objective**: To evaluate the effects of N-PEP-12 supplementation on attention, cognitive function, and mental wellbeing in healthy middle-aged and older adults with SCCs. **Methods**: In this prospective, randomized, double-blind, placebo-controlled trial, 276 participants aged 50–75 years with SCCs and no clinically significant cognitive impairment were randomized to placebo, N-PEP-12 45 mg, or N-PEP-12 90 mg. Assessments were performed at baseline and after 30, 90, and 180 days. Primary outcomes included Test of Attentional Performance measures. Secondary outcomes included WAIS-IV Digit Span Forward and Backward, perceived stress, mood, sleep quality, and EQ-5D-5L visual analog scale. **Results**: Across the three primary attention outcomes analyzed jointly in a multivariate repeated-measures model, there was a significant group-by-visit interaction (*p* = 0.003) and a significant effect of visit (*p* < 0.001), without a significant main effect of treatment arm (*p* = 0.133), indicating a time-dependent treatment effect. This result was obtained in a sensitivity analysis population in which missing values were imputed under assumptions least favorable to the active arms. In exploratory endpoint-specific comparisons at 90 days, both N-PEP-12 groups showed greater improvements than placebo in alertness, attention omissions and memory omissions, and Digit Span Forward and Backward scores also improved. In a subsequent uncontrolled extension phase, in which all participants received active treatment, participants initially assigned to placebo showed comparable improvements after switching to N-PEP-12 90 mg. These observations are exploratory. Adverse events were infrequent and similarly distributed across groups. **Conclusions**: N-PEP-12 supplementation was associated with improvements in attention, working memory, and mental wellbeing in healthy middle-aged and older adults with subjective cognitive complaints. These findings support further investigation of N-PEP-12 as a nutritional intervention for early subjective cognitive changes associated with aging.

## 1. Introduction

The global population is aging at a high rate, with the number of adults aged 60 and over expected to grow rapidly in the coming decades [1]. As people live longer, maintaining cognitive health and mental wellbeing has become increasingly important for both individuals and healthcare systems. Healthy aging is not defined just by the absence of disease, but also involves preserving the cognitive, emotional, and functional abilities that allow people to remain independent and maintain a good quality of life [2]. Therefore, identifying safe and accessible interventions that may help sustain cognitive performance in later life is becoming more important than ever.

With aging, gradual changes across different cognitive areas are common, even among otherwise healthy individuals. Cross-sectional and longitudinal studies suggest that attention, working memory, and processing speed tend to decline progressively from midlife onward, which can affect everyday functioning and overall wellbeing [3,4,5]. These changes often become noticeable when individuals begin to experience subtle cognitive difficulties, even before any objective impairment can be detected or diagnosed. Subjective cognitive complaints (SCC) are therefore common in middle-aged and older adults and are gaining recognition as meaningful clinical signals [6]. Even in healthy individuals, such experiences may reflect early shifts in cognitive efficiency, greater mental effort during tasks, or even a reduced sense of confidence in one’s abilities. All these factors can greatly affect quality of life [6,7].

Subjective cognitive complaints are often linked to broader aspects of mental wellbeing, such as stress, sleep and mood. These factors may both influence and be influenced by these cognitive difficulties, creating a complex interaction which can affect daily life and wellbeing [8,9,10,11,12].

N-PEP-12 is a peptide-based nutritional supplement that has shown promising neuroprotective effects in both experimental and early clinical studies [13,14,15]. It was reported to protect neurons from various types of damage in a dose-dependent manner, making it a candidate for treating ischemic stroke and other neurodegenerative disorders [15,16]. However, when it comes to its potential effects in healthy individuals, especially those experiencing subjective cognitive complaints, the available evidence remains limited. Most existing studies have focused on populations with established cognitive impairment, leaving an important gap in understanding if and how N-PEP-12 may be beneficial in earlier stages of cognitive change. N-PEP-12 is obtained from purified porcine brain proteins and is a derivative of the parent peptide preparation Cerebrolysin. In post-stroke populations, its supplementation has been associated not only with improvements on neuropsychological testing, but also with measurable changes in cortical bioelectrical activity on quantitative electroencephalography, suggesting an effect at the level of network-scale brain function rather than test performance alone [16,17].

Interest in N-PEP-12 sits within a broader body of work on the nutritional modulation of brain function. Dietary and endogenous small molecules can influence neurotransmission and cerebral energy metabolism through several converging routes, including direct modulation of cholinergic enzyme activity, provision of substrates for neurotransmitter synthesis, and effects on oxidative and inflammatory tone [18]. Amino acid metabolism, in particular, appears to be altered in cognitive aging, and circulating amino acid profiles have been proposed as candidate biomarkers distinguishing older adults with dementia from those without dementia [19]. Because N-PEP-12 consists of low-molecular-weight peptides together with free amino acids, both peptide-mediated and amino-acid-mediated mechanisms are plausible contributors to any observed effect, and this framing informs the interpretation of our findings.

The present study explored the potential of N-PEP-12 dietary supplementation in enhancing attentional performance, cognition, and mental wellbeing in healthy middle-aged and older adults with subjective cognitive complaints. Specifically, this study assessed both objective cognitive outcomes and self-reported measures of stress, mood, sleep quality, and health-related quality of life.

## 2. Materials and Methods

### 2.1. Study Procedures

This was a prospective, randomized, placebo-controlled, double-blind study conducted at the “RoNeuro” Institute for Neurological Research and Diagnostic in Cluj-Napoca, Romania between 2023 and 2025. The study assessed the efficacy of N-PEP-12 supplementation on attentional performance, cognitive function, and mental wellbeing in healthy middle-aged and older adults with subjective cognitive complaints, with primary evaluation at 90 days and additional assessments at 30 and 180 days.

Participants aged between 50 and 75 years were eligible for recruitment if they reported SCCs but did not show evidence of clinically significant cognitive impairment, with a Montreal Cognitive Assessment score > 25.

The trial received approval from the Ethics Committee of the Iuliu Hatieganu University of Medicine and Pharmacy (approval date: 28 November 2022) and was conducted in accordance with the Declaration of Helsinki. The study was prospectively registered in the ISRCTN (International Standard Randomized Controlled Trial Number) registry (ISRCTN10514154; registered on 3 April 2023; doi: https://doi.org/10.1186/ISRCTN10514154). All participants provided signed informed consent before participation. The trial was conducted in accordance with the prospectively registered protocol and statistical analysis plan.

### 2.2. Inclusion and Exclusion Criteria

Participants were eligible for inclusion if they met the following criteria: age between 50 and 75 years (inclusive), ability to understand the study procedures and provide written informed consent, and sufficient literacy to complete the neuropsychological assessments. Eligible participants were required to have a body weight of at least 50 kg for males and 45 kg for females, with a body mass index (BMI) between 18.0 and 29.9 kg/m^2^. All participants were in generally good health, as confirmed by clinical examination and laboratory testing, and had no clinically significant cognitive impairment, defined as a Montreal Cognitive Assessment (MoCA) score greater than 25. Participants were required to report subjective cognitive complaints.

Participants were excluded if they had a history of alcohol abuse (defined as more than 14 units per week) or drug abuse within the five years preceding screening or if they tested positive on urine drug screening. Individuals who had participated in another clinical trial within three months prior to enrollment were also excluded. Further exclusion criteria included the presence of clinically significant systemic diseases, such as neurological, psychiatric, cardiovascular, endocrine, or oncological conditions, as well as a history of major neurological disease. Participants with a history of bariatric surgery (such as gastric sleeve) were excluded. Finally, individuals with functional impairments that could interfere with cognitive testing, such as injury to the dominant hand, were not eligible for participation.

### 2.3. Study Assessments

Cognitive performance was assessed using the Test of Attentional Performance version 2.3.1 (TAP) (Psytest, Herzogenrath, Germany) as a primary variable, including three subtests: alertness (phasic alertness), which measures reaction time to auditory warning cues; working memory (difficulty level 3), which evaluates the ability to update and retain information under higher cognitive demands; and divided attention, which examines accuracy and reaction times during simultaneous task performance [20].

Additionally, the Wechsler Adult Intelligence Scale-Fourth Edition (WAIS-IV) was used as a secondary variable, specifically the Digit Span Forward (DSF) and Digit Span Backward (DSB) subtests, to assess short-term memory and working memory [21]. In addition, several validated self-report measures were also administered to assess broader aspects of mental wellbeing. Perceived stress was evaluated using the Perceived Stress Scale (PSS) [22], while mood was assessed with the Brief Mood Introspection Scale (BMIS) [23]. Sleep quality was measured using the Sleep Quality Scale (SQS) [24] and overall health-related quality of life was assessed using the EQ-5D-5L instrument, including its visual analog scale component [25].

### 2.4. Study Visits

At the first study visit (Visit 1, Day 0), participants underwent screening and baseline assessments. After being informed about the study procedures, all participants provided written informed consent. Eligibility was confirmed based on the predefined inclusion and exclusion criteria, after which participants were randomized to one of the three study groups (placebo, 45 mg N-PEP-12 or 90 mg N-PEP-12). Demographic information, medical history, and relevant risk factors were recorded, along with the initial assessment of primary and secondary outcome measures. At Day 30, participants returned for the first follow-up evaluation, which included reassessment of outcome measures and monitoring of treatment adherence and safety. At Day 90, another efficacy evaluation was conducted. Following this visit, participants originally assigned to the placebo group were switched to 90 mg N-PEP-12, while those in the active treatment groups continued their initial dosing regimen. At the last study visit (Day 180), participants underwent re-evaluation of outcome measures, along with continued safety and adherence monitoring. During the period between Visit 3 and Visit 4, all participants received active treatment, either continuing their assigned N-PEP-12 dose or, in the case of the initial placebo group, receiving 90 mg N-PEP-12. Adverse events were collected non-systematically by open questioning at each study visit and recorded.

### 2.5. Study Product

N-PEP-12 (marketed as MemoProve^®^ and Cebrium^®^) was supplied by EVER Neuro Pharma GmbH (Unterach am Attersee, Austria). N-PEP-12 is not a single defined molecular entity. It is a proprietary preparation derived from the standardized enzymatic proteolysis of purified porcine brain proteins and consists of a heterogeneous mixture of low-molecular-weight peptides together with free amino acids, being a derivative of the parent peptide preparation Cerebrolysin, produced by the same manufacturer. The product was administered orally as film-coated tablets containing lactose monohydrate as a carrier together with standard pharmaceutical excipients. Participants in the two active arms received one tablet daily, providing 45 mg or 90 mg of N-PEP-12 according to allocation, for the full duration of the study. Placebo units were manufactured to be indistinguishable from active product in size, shape, color, weight, and organoleptic properties and were supplied in identical blisters and outer cartons.

Batch-to-batch consistency is ensured by the manufacturer through a standardized manufacturing process and release testing against predefined specifications. The detailed peptide and free amino acid composition, the molecular-weight distribution, and the quality-control specifications of the preparation are proprietary to the manufacturer and were not disclosed to the investigators. All study medication was supplied in unlabelled white cartons bearing only the randomization code, in order to preserve blinding, and individual batch numbers were not recorded at participant level.

### 2.6. Randomization and Blinding

Participants were randomly assigned in a 1:1:1 ratio to placebo, N-PEP-12 45 mg, or N-PEP-12 90 mg using permuted block randomization (block size of 3), in accordance with ICH E9 guidance on statistical principles for clinical trials. The randomization list was generated within the study’s electronic case report form (eCRF) by the study data manager, who had no role in participant recruitment or assessment.

Allocation concealment was ensured through a sealed random code list together with a corresponding set of sealed, sequentially numbered envelopes, to be opened only in the event of a medical emergency requiring unblinding. Medication was dispensed to participants throughout the trial, including after the Day 90 switch, by a dedicated study nurse who was not involved in outcome assessment. Outcome assessors remained unaware of participants’ treatment allocation throughout the study, including during the Day 90–180 extension period. Treatment adherence was assessed at each visit by counting returned empty packaging. Concomitant medication and supplement use was reviewed at each visit, with participants asked to report any additions to their treatment regimen since the prior visit. No concomitant medications or supplements with a potential interaction with the study product were reported.

### 2.7. Sample Size Calculation

The sample size was determined for a three-arm trial (placebo and two active treatment arms, K = 2 experimental treatments vs. control) using a Dunnett-type multiple-comparison procedure, with all pairwise comparisons made against the placebo arm. The significance level was set at α = 0.05 (two-sided), with the family-wise error rate controlled at 0.05 across the two experimental-versus-control comparisons. Minimum marginal power was set at 1 − β = 0.90, controlled under each comparison’s least favorable configuration. The standardized effect sizes assumed for the interesting and uninteresting treatment differences were δ_1_ = 0.5 and δ_0_ = 0, respectively, with a common standard deviation across arms (σ_0_ = … = σ_k_ = 1). The target allocation ratio for each experimental arm relative to the control arm was 1:1, giving a realized allocation ratio of (r_1_, r_2_) = (1, 1) and an equal per-arm sample size. Under these assumptions, the adjusted critical threshold for the pairwise comparisons was 0.028. Accounting for an anticipated 10% dropout rate, the minimum required total sample size was *n* = 270, corresponding to 90 participants per arm (n_0_ = n_1_ = n_2_ = 90). The calculation was performed using the web application of Grayling and Wason for the design of multi-arm clinical trials [26].

### 2.8. Data Analysis

Statistical analyses were performed using R version 4.5.2 [27] (R Foundation, Vienna, Austria) and RStudio version 2025.09.2+418 (Posit Software, PBC, Boston, MA, USA), utilizing the renv (v1.1.7) [28], openxlsx (v4.2.8.1) [29], stringr (v1.6.0) [30], effectsize (v1.0.2) [31], ggplot2 (v4.0.2) [32] and MANOVA.RM (v0.5.4) [33] packages. Efficacy was evaluated in both Intention-to-Treat (ITT) and per-protocol (PP) populations, with missing data managed via pairwise and listwise deletion, respectively, according to the pre-specified Statistical Analysis Protocol (SAP); additionally, the sensitivity analysis (SA) was done using a Missing Not at Random (MNAR) framework, where missing data was imputed either as most favorable scores for the placebo group, or as least favorable scores for the N-PEP-12 treated group (most and least favorable scores observed across the trial cohort for each endpoint respectively). The sensitivity analysis population comprised all randomized participants (*n* = 276; 92 per arm), with missing post-randomization values imputed within this framework so that complete data were available at every study visit. This analysis was performed in addition to the ITT and PP analyses in order to assess the robustness of the primary findings to a strongly adverse departure from the missing-at-random assumption. Where the Test of Attentional Performance returned T-scores below the instrument floor of 20, these values were recoded to 19 prior to analysis in order to preserve the ordinal ranking of below-floor performance without introducing implausible negative values. To evaluate longitudinal improvements across the second and third visits while avoiding parametric distribution assumptions and multiple testing, primary efficacy across the TAP battery (alertness, attention omissions and memory omissions T-scores) was analyzed using a multivariate non-parametric repeated-measures model (the multRM function in the MANOVA.RM package, reporting resampling-based modified ANOVA-type statistic *p*-values using 10,000 resampling iterations) based on the SA population. For secondary outcomes, we also reported resampling-based modified ANOVA-type statistic *p*-values determined using the function RM of the MANOVA.RM package. For further comparisons, continuous data normality was assessed using the Shapiro–Wilk test. Baseline was incorporated by analyzing change scores: for each outcome, the model was fitted to the differences from the first visit (V2 − V1 and V3 − V1), so that the visit infactor comprised two levels and each participant’s baseline value served as their own reference. The confirmatory model was restricted to the randomized, placebo-controlled phase, and changes at Day 180 (V4 − V1) are reported descriptively as part of the uncontrolled extension. Between-group differences were analyzed via Student’s *t*-test or Wilcoxon tests based on normality, while categorical data were compared using Pearson’s chi-square test where all expected cell counts were ≥5, and the Fisher–Freeman–Halton exact test otherwise. Within-group changes were evaluated using paired Student’s *t*-tests or Wilcoxon signed-rank tests. Effect sizes were reported as Cohen’s d parametric for unpaired and paired data), Cliff’s δ (non-parametric for unpaired data) and rank-biserial correlation *r*_rb_ (non-parametric for paired data). Thresholds for Cohen’s d were interpreted as small (d = 0.2), medium (d = 0.5) and large (d = 0.8) based on Cohen (1988) [34]. For Cliff’s δ, thresholds were defined as small (δ = 0.147), medium (δ = 0.33) and large (δ = 0.474) in accordance with Romano et al. (2006) [35]. Rank-biserial correlations were also interpreted according to Cohen’s thresholds for correlation coefficients as small (*r*_rb_ = 0.1), medium (*r*_rb_ = 0.3), and large (*r*_rb_ = 0.5) [34]. Outcome data were represented as mean ± standard deviation of differences to baseline values from the ITT population to ensure intelligibility. Safety was assessed via risk ratios, odds ratios and Number Needed to Harm (NNH). All tests were two-tailed with significance set at *p* < 0.05. Between-group, within-group and secondary outcome analyses were considered exploratory in nature.

## 3. Results

**Figure 1:**
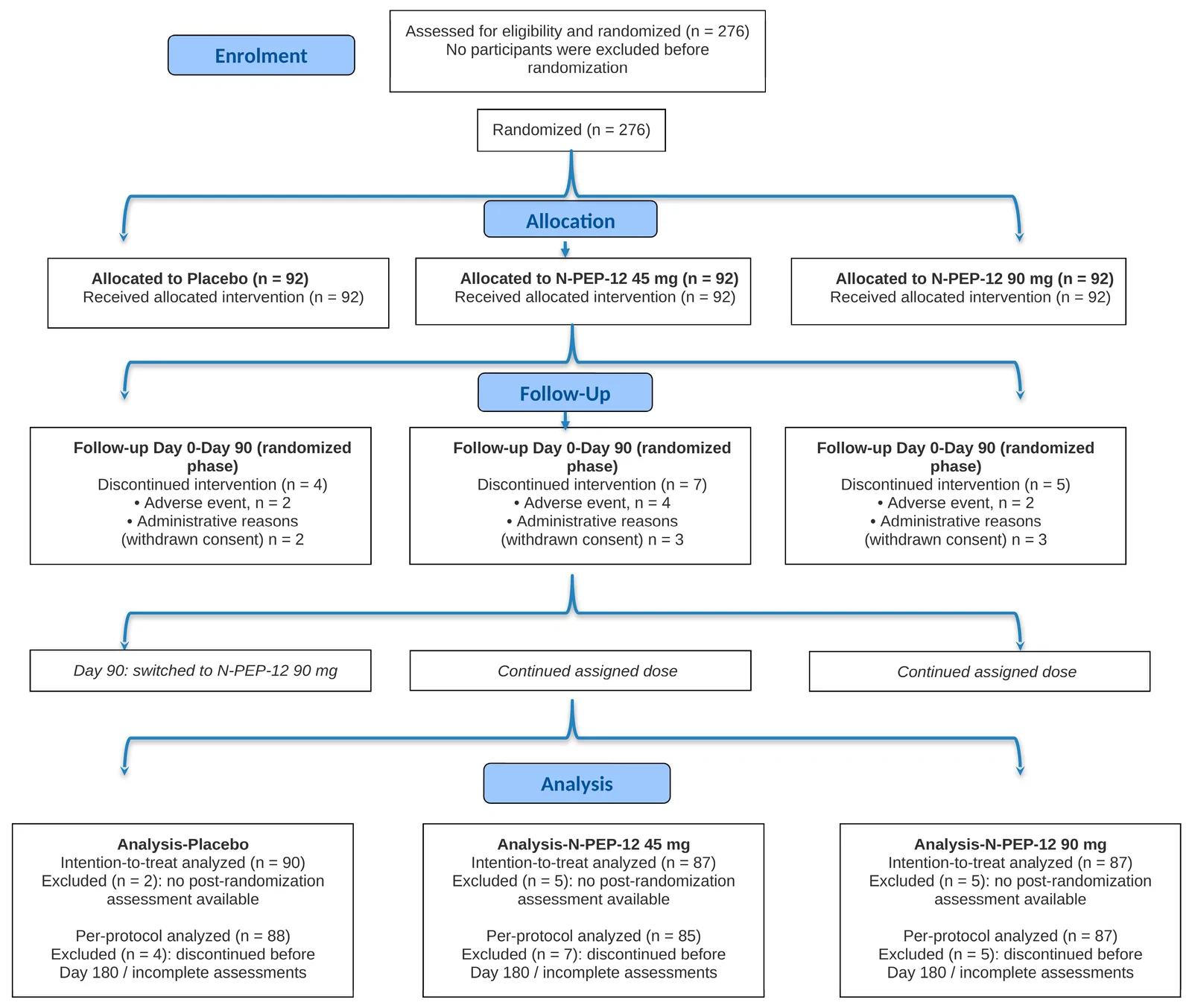
CONSORT flow diagram of participant enrollment, randomization, allocation, follow-up and analysis populations. All 276 participants assessed for eligibility met inclusion criteria and were randomized; no participants were excluded before randomization. Reasons for discontinuation during the randomized phase (Day 0–Day 90) are reported by group. At Day 90, participants originally allocated to placebo were switched to N-PEP-12 90 mg, while participants in the two active arms continued their assigned dose. Intention-to-treat (ITT) and per-protocol (PP) analysis populations are reported separately for each arm, with reasons for exclusion from each.

The study enrolled a total of 276 subjects, equally distributed across the three arms (*n* = 92 per arm; Figure 1). Discontinuation rates did not differ significantly between groups (Chi^2^ *p* = 0.628). Outcome analysis was performed for the intention-to-treat (ITT) population (*n* = 264 contributing at least one post-baseline assessment; the number contributing at each visit declines at later visits under pairwise deletion), with per-protocol (PP) results (*n* = 260, nearly identical) available in the Supplementary Material (Supplementary_Outcomes). Participant flow through enrollment, randomization, follow-up and analysis is shown in Figure 1.

The mean age at enrollment was 61.4 ± 7.4 years (range, 50 to 75 years), and the majority of participants were females (199, 72.1%). Baseline demographic characteristics (Table 1) and outcome measures (Table 2) were similar across the three groups.

### 3.1. Primary Outcomes

The primary conclusions of this trial are supported by the multivariate repeated-measures model fitted to the sensitivity analysis population (*n* = 276), in which missing values were imputed under assumptions least favorable to the active arms. The intention-to-treat population (*n* = 264) supports the descriptive and exploratory analyses reported below and is the population shown in all figures. The per-protocol population (*n* = 260) was analyzed as a supporting analysis. The multivariate model requires complete follow-up for all included participants and can therefore be fitted only to the per-protocol and sensitivity analysis populations.

The primary outcomes were assessed at the 90-day visit and compared to baseline values. In addition, changes in TAP within the placebo group were examined before and after switch to the 90 mg treatment regimen. Outcomes in the intervention groups (45 mg and 90 mg) were compared against the placebo group to account for potential improvements related to repeated exposure to the test battery.

Using the multivariate mixed-model semi-parametric MANOVA.RM model, there was a statistically significant effect of the group-visit interaction (*p* = 0.003) and of the visit within-group factor (*p* < 0.001), with no statistically significant effect of the treatment arm as a between-group factor (*p* = 0.133), over the response across the three main outcomes (TAP T-scores for alertness, attention and memory). Thus, even under disadvantageous data imputation strategies, there is a statistically significant time-dependent therapeutic effect of N-PEP-12 over placebo. Moreover, in the per-protocol population, the same model revealed statistically significant effects for the group-visit interaction, the visit term, as well as the group term, highlighting the effect of N-PEP-12 even at the second visit (after 30 days of treatment) compared to placebo.

Comparisons involving the Day 90 to Day 180 interval require a separate interpretive framework. From Day 90 onward, all participants received active treatment and no concurrent placebo group remained, so these comparisons are uncontrolled and are reported here as exploratory. Within-participant changes observed in the group switched from placebo to N-PEP-12 90 mg cannot be attributed to the intervention with the same confidence as the randomized Day 0 to Day 90 comparisons, since period effects, repeated exposure to the test battery, regression to the mean, and changes in participant expectation following the protocol-specified switch may all contribute.

Regarding alertness, both the N-PEP-12 45 mg group (Wilcoxon rank-sum test, *p* < 0.001, δ = 0.405 CI 95% [0.252 to 0.538]) and the 90 mg group (*p* < 0.001, δ = 0.446 CI 95% [0.3 to 0.572]) demonstrated significantly greater improvements compared to placebo. Following crossover to the high-dose regimen, participants initially assigned to placebo showed a significant increase in alertness relative to their prior trajectory (paired Student *t*-test *p* < 0.001, d = 0.563 [0.337 to 0.787]). Effect size estimates (r_rb_) indicated large effects for both N-PEP-12 doses after 90 days of treatment. At the final assessment, no significant differences were observed between participants who received 90 mg N-PEP-12 after crossover and those treated continuously for 180 days (either 45 mg or 90 mg), whether considering absolute scores or changes from baseline.

**Figure 2:**
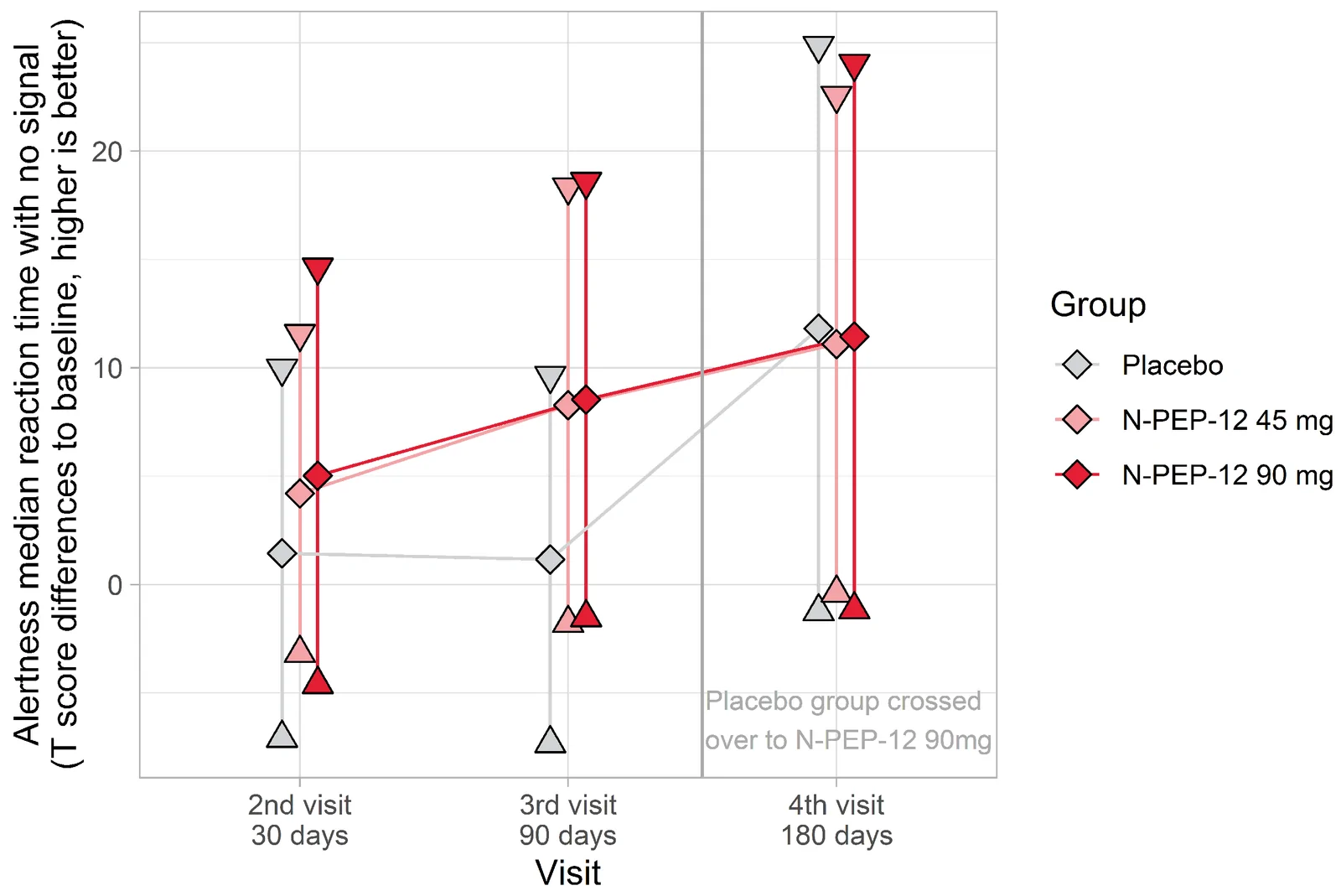
Distribution of alertness T-score from median reaction time with no signal. The diamond represents the mean, and inward triangles represent the standard deviation referenced against the mean. Data from the Intention-To-Treat (ITT) population.

In this uncontrolled phase, no significant differences were observed between participants switched to 90 mg at Day 90 and those treated continuously for 180 days. This observation is compatible with a plateau in effect beyond 90 days, but in the absence of a concurrent control group it should be regarded as exploratory and hypothesis-generating only. These results are illustrated in Figure 2. 

**Figure 3:**
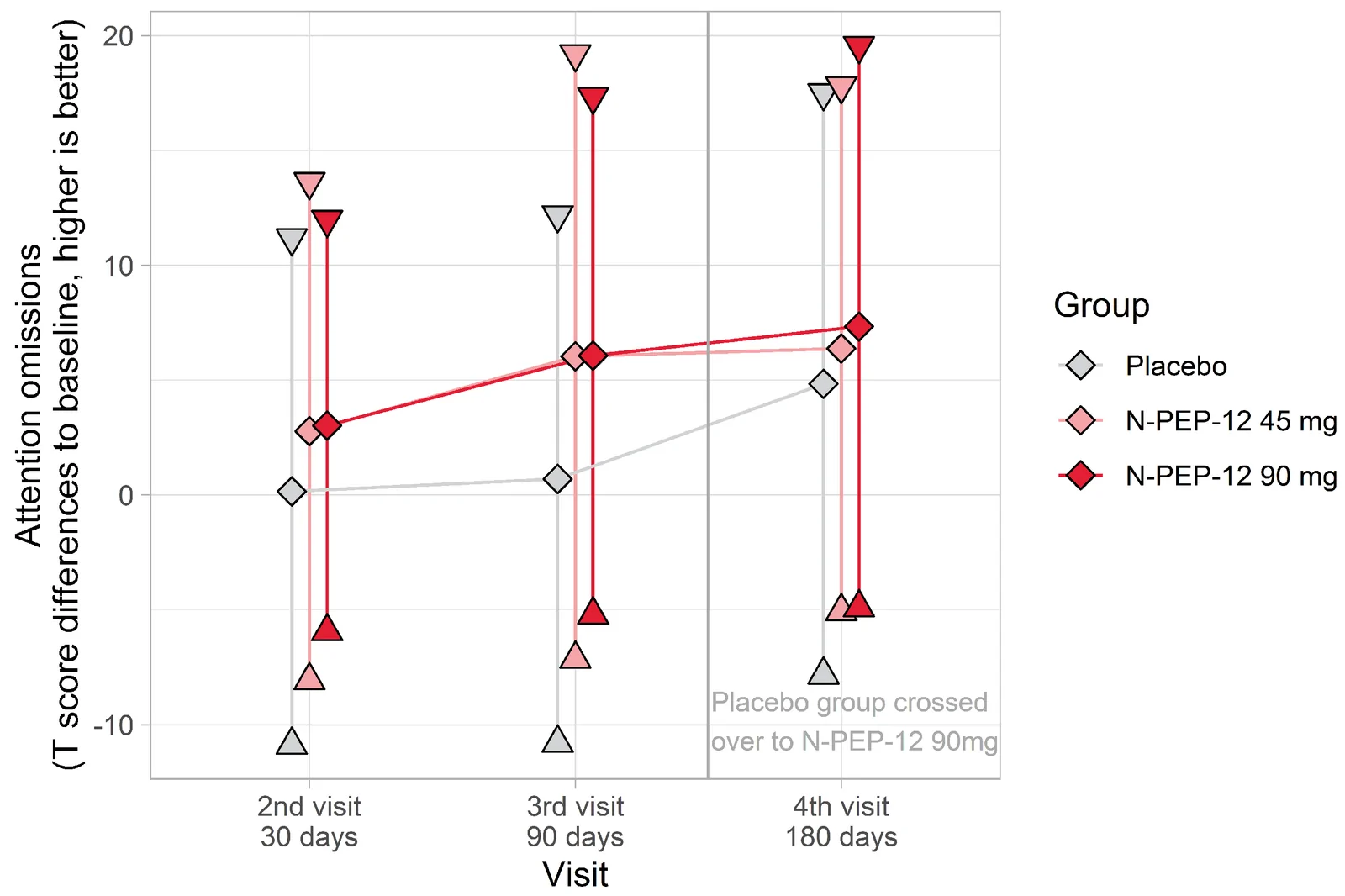
Distribution of attention T-score based on the number of omissions. The diamond represents the mean, and inward triangles represent the standard deviation referenced against the mean. Data from the Intention-To-Treat (ITT) population.

Likewise, we observed an improvement in the number of omissions during the task. The placebo group did not show a significant change over time (Wilcoxon signed-rank test *p* = 0.491, *r*_rb_ = 0.091 [−0.147 to 0.319]), suggesting minimal practice effects. In contrast, both the 45 mg (Wilcoxon rank-sum test *p* = 0.005, δ = 0.248 [0.081 to 0.401]) and 90 mg (*p* = 0.002, δ = 0.266 [0.101 to 0.417]) N-PEP-12 groups demonstrated significantly greater improvements compared to placebo. Following crossover, participants initially assigned to placebo did not show a significant benefit after switching to the high-dose regimen (paired Student’s *t*-test, *p* = 0.143, d = 0.158 [−0.053 to 0.368]). The corresponding distributions are shown in Figure 3.

**Figure 4:**
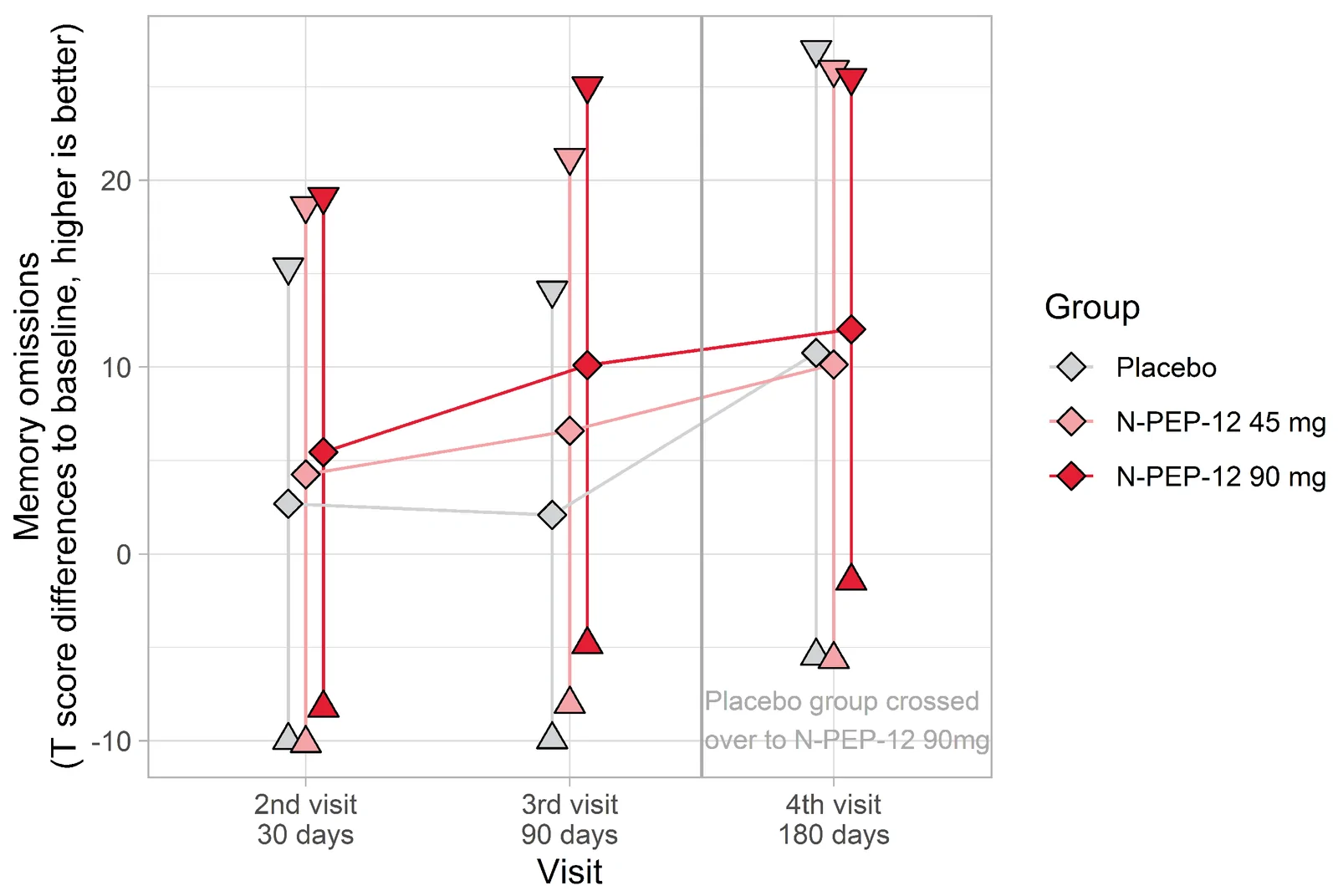
Distribution of memory T-score based on the number of omissions. The diamond represents the mean, and the inward triangles represent the standard deviation referenced against the mean. Data from the Intention-To-Treat (ITT) population.

Lastly, for the number of omissions in the memory task, the placebo group showed a slight but statistically non-significant improvement over time (Wilcoxon signed-rank test *p* = 0.057, *r*_rb_ = 0.27 [0.038 to 0.475]), likely reflecting practice effects. In contrast, both the low-dose (45 mg; *p* = 0.012, δ = 0.219 [0.051 to 0.375]) and high-dose (90 mg; *p* < 0.001, δ = 0.321 [0.16 to 0.465]) N-PEP-12 groups demonstrated significantly greater improvements compared to placebo. Following crossover, participants initially assigned to placebo showed a significantly greater improvement compared to their own prior performance (paired Student *t*-test, *p* = 0.005, d = 0.309 [0.094 to 0.522]). This improvement was comparable to that observed in participants who received 90 mg N-PEP-12 from baseline (Wilcoxon rank-sum test, *p* = 0.627, δ = −0.042 [−0.211 to 0.128]). These results are illustrated in Figure 4.

### 3.2. Secondary Outcomes

#### 3.2.1. Wechsler Adult Intelligence Scale

**Figure 5:**
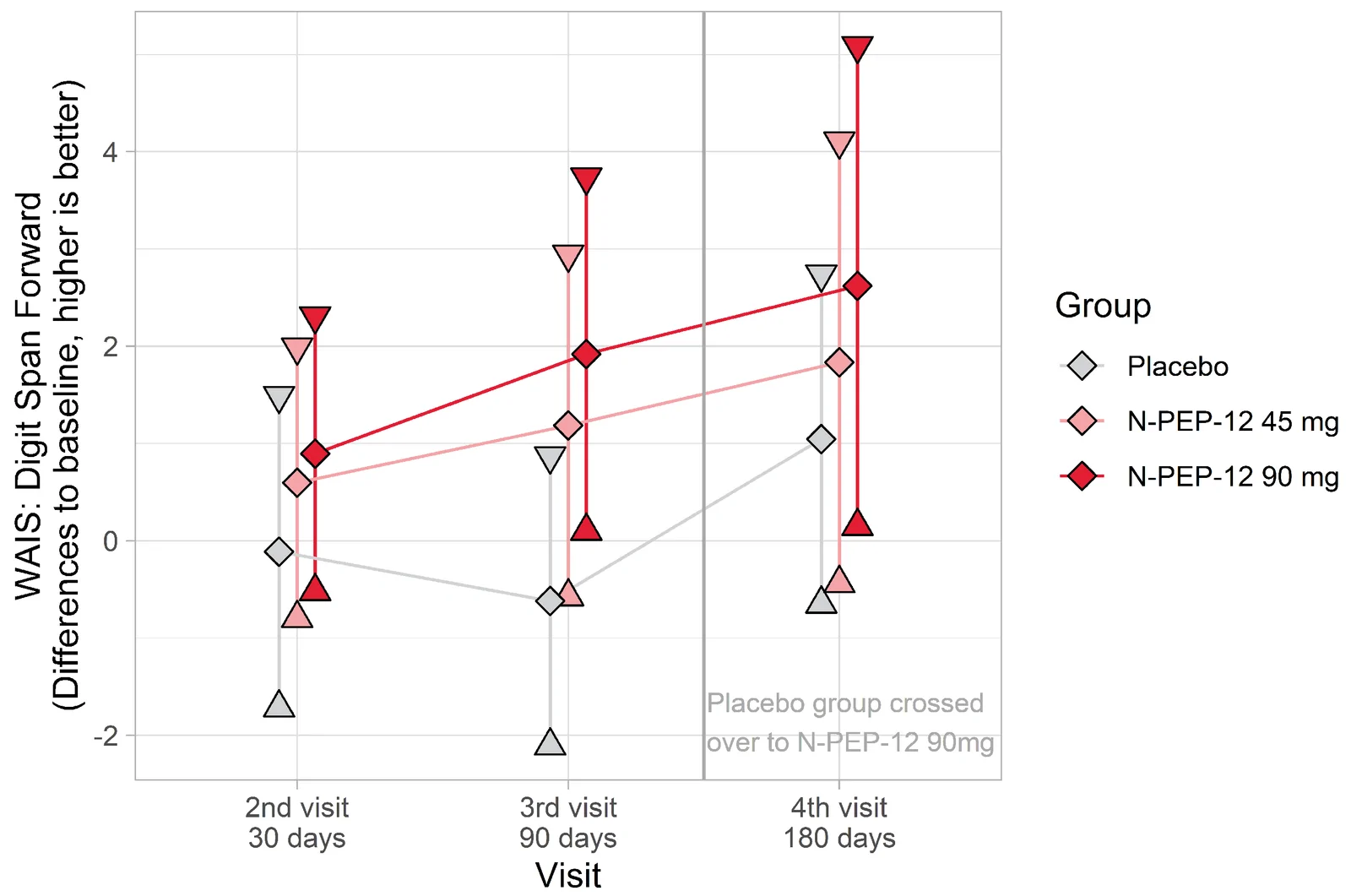
Distribution of Digit Span Forward values, part of the Wechsler Adult Intelligence Scale. The diamond represents the mean, and the inward triangles represent the standard deviation referenced against the mean. Data from the Intention-To-Treat (ITT) population.

Regarding the Digit Span Forward task of the Wechsler Adult Intelligence Scale, the placebo group showed a decline in performance at 90 days compared to baseline (Wilcoxon signed-rank test, *p* < 0.001, *r*_rb_ = −0.496 [−0.655 to −0.296]). In contrast, both treatment groups demonstrated significant improvements over time (Wilcoxon signed-rank test, *p* < 0.001 for both the 45 mg and 90 mg N-PEP-12 groups), with moderate to large gains compared to the placebo group (Wilcoxon rank-sum test, *p* < 0.001 for both groups; δ = 0.571 [0.444 to 0.675] for 45 mg and δ = 0.703 [0.606 to 0.78] for 90 mg). After the crossover at 90 days, the placebo-initiated participants showed improvements compared to their prior trajectory (paired Student *t*-test *p* < 0.001, d = 0.885 [0.636 to 1.13]), obtaining a performance comparable to the subjects who were initiated with 90 mg (Wilcoxon rank-sum test *p* = 0.372, δ = −0.077 [−0.244 to 0.094]). The sensitivity analysis revealed statistically significant effects for all analyzed factors: trial arm (between-group factor), visit (within-group factor), and their interaction (*p* < 0.001), highlighting the effect of N-PEP-12 on improved performance during this task. The corresponding distributions are shown in Figure 5.

**Figure 6:**
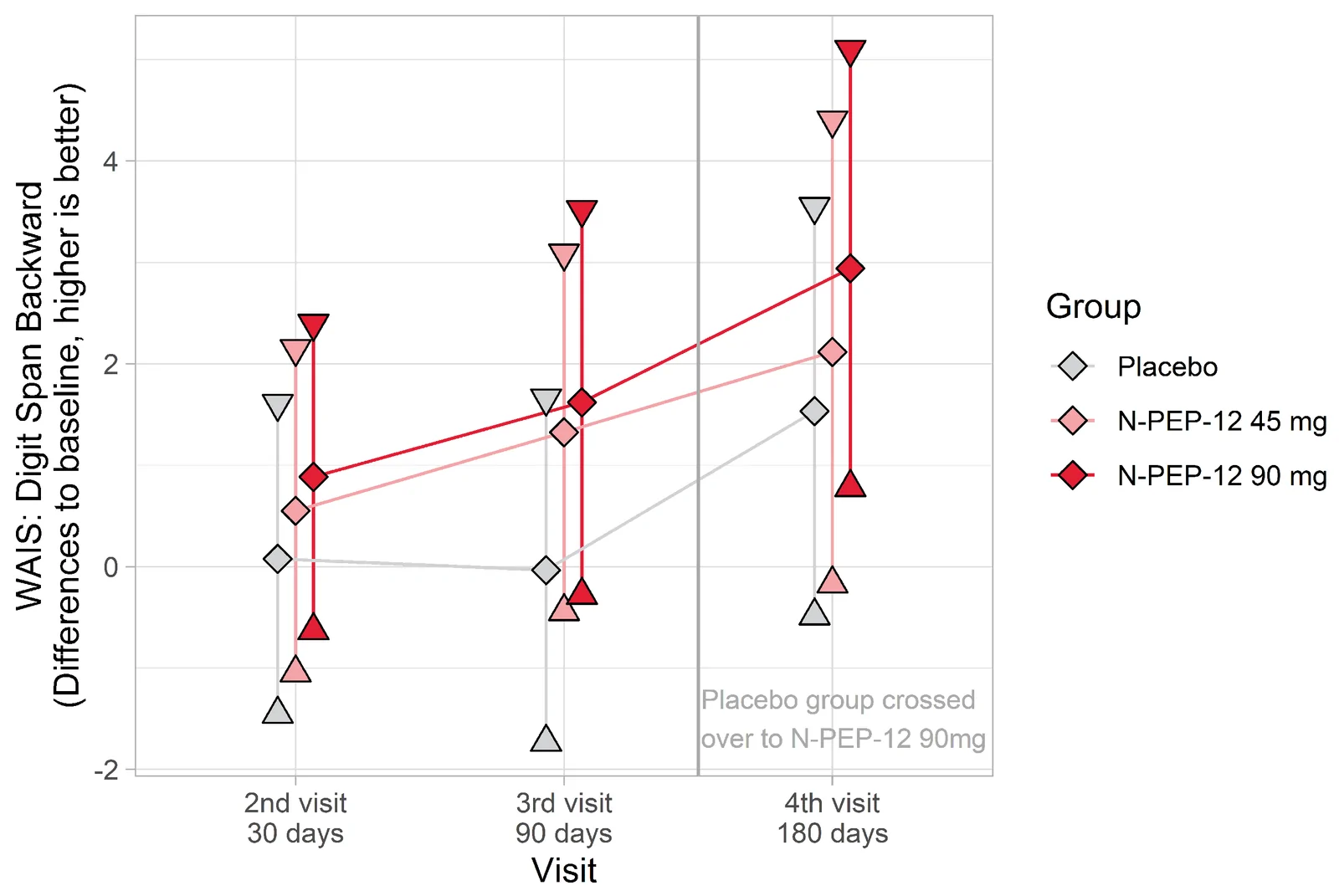
Distribution of Digit Span Backward values, part of the Wechsler Adult Intelligence Scale. The diamond represents the mean, and the inward triangles represent the standard deviation referenced against the mean. Data from the Intention-To-Treat (ITT) population.

For the Digit Span Backward task, the performance of the placebo group remained constant (Wilcoxon signed-rank test *p* = 0.852, *r*_rb_ = −0.028 [−0.261 to 0.208]) and both groups treated with low and high-dose N-PEP-12 observed statistically significant improvements of moderate effect size (Wilcoxon rank-sum test *p* < 0.001, δ = 0.408 [0.255 to 0.54] for 45 mg and *p* < 0.001, δ = 0.472 [0.329 to 0.594] for 90 mg). After crossover, the placebo-initiated group observed significant improvement of moderate effect size (Wilcoxon signed-rank test *p* < 0.001, *r*_rb_ = 0.581 [0.387 to 0.726]), improvement being comparable to the one observed by the group initiated on 90 mg dosage (Wilcoxon rank-sum test *p* = 0.979, δ = 0.002 [−0.168 to 0.172]). The sensitivity analysis revealed statistically significant effects for the visit as a within-group term (*p* < 0.001) and for the group-visit interaction (*p* = 0.003), with a tendency towards statistical significance for the treatment arm (between groups) factor (*p* = 0.082), highlighting a time-dependent effect. These results are illustrated in Figure 6.

#### 3.2.2. Mental Wellbeing and Quality of Life

**Figure 7:**
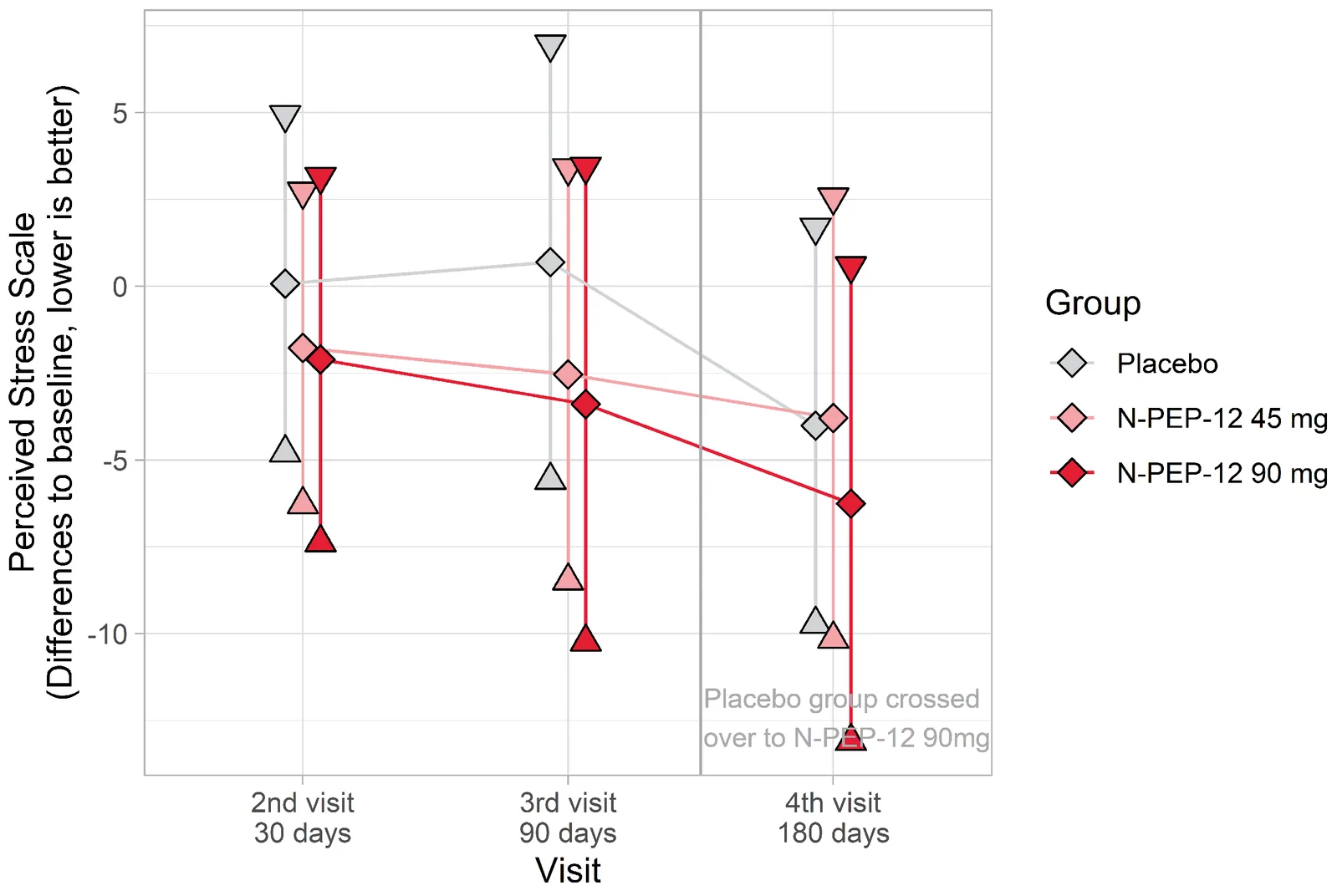
Distribution of Perceived Stress Scale scores. The diamond represents the mean, and the inward triangles represent the standard deviation referenced against the mean. Data from the Intention-To-Treat (ITT) population.

Because lower scores on the Perceived Stress Scale indicate reduced stress, negative differences (including effect sizes) reflect improvement. The placebo group did not show a significant change up to the third visit (paired Student *t*-test *p* = 0.298, d = 0.111 [−0.098 to 0.319]). In contrast, both the 45 mg (Wilcoxon rank-sum test, *p* < 0.001, δ = −0.32, 95% CI [−0.465 to −0.159]) and 90 mg (*p* < 0.001, δ = −0.412, 95% CI [−0.543 to −0.26]) N-PEP-12 groups demonstrated significantly greater improvements compared to placebo, with small to moderate effect sizes. Following crossover, participants initially assigned to placebo showed a significant improvement compared to their prior trajectory (paired Student *t*-test *p* < 0.001, d = −0.544 [−0.767 to −0.319]). This improvement was comparable to that observed in participants who received 90 mg N-PEP-12 from baseline (Wilcoxon rank-sum test, *p* = 0.414, δ = −0.071, 95% CI [−0.238 to 0.1]). In the sensitivity analysis, Perceived Stress Scale scores were not influenced by any of the three factors: treatment group (*p* = 0.307), visit (*p* = 0.18), or group-visit interaction (*p* = 0.119). The corresponding distributions are shown in Figure 7.

**Figure 8:**
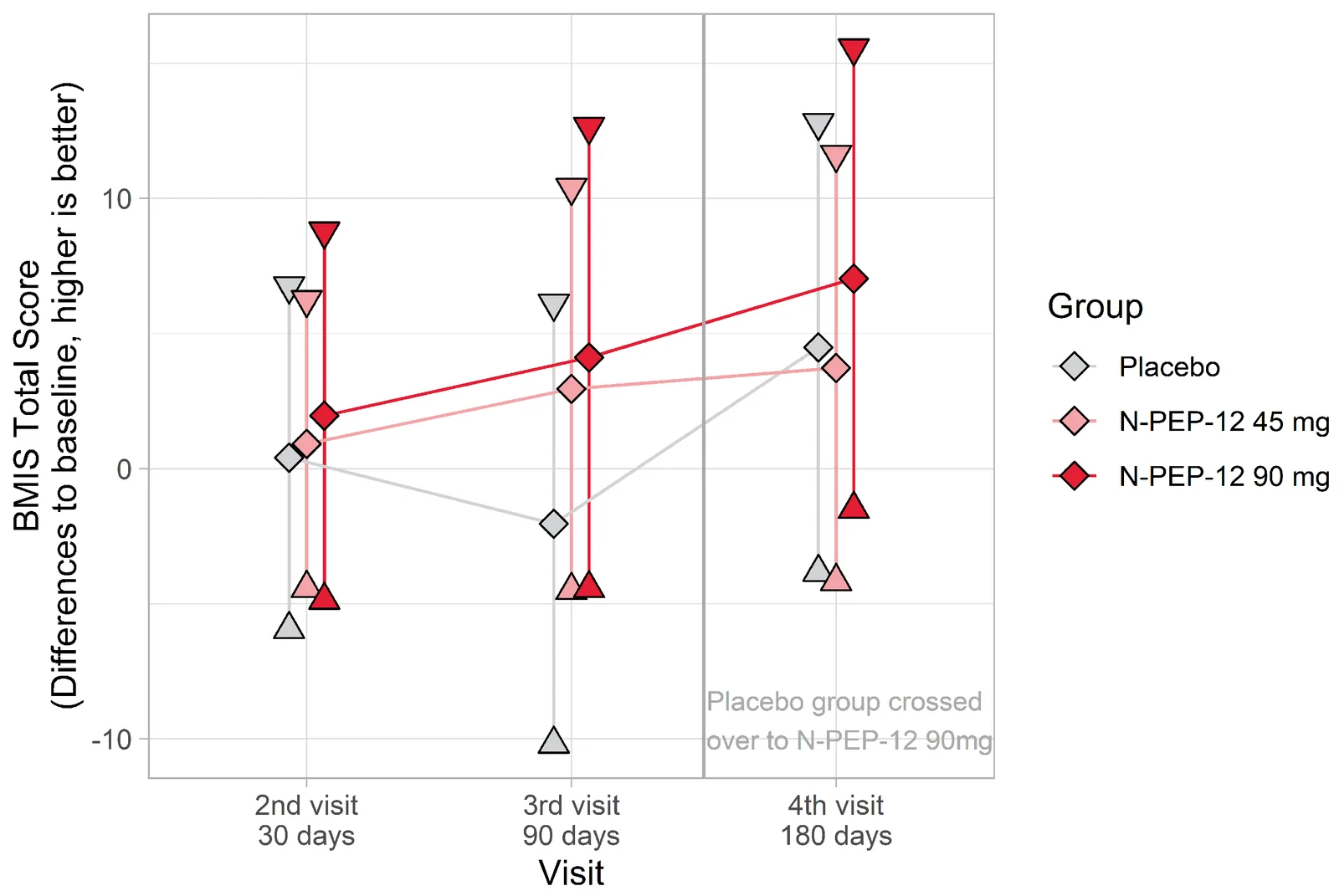
Distribution of Brief Mood Introspection Scale (BMIS) total scores. The diamond represents the mean, and the inward triangles represent the standard deviation referenced against the mean. Data from the Intention-To-Treat (ITT) population.

Regarding the Brief Mood Introspection Scale (BMIS), subjects in the placebo group showed a slight decline in scores at 90 days compared to baseline (paired Student *t*-test *p* = 0.021, d = −0.25 [−0.46 to −0.038]). In comparison, both treatment groups had higher scores, with moderate to great improvements at 90 days compared to the placebo group (Wilcoxon rank-sum test *p* < 0.001 for both groups, δ = 0.389 [0.234 to 0.524] for 45 mg and δ = 0.469 [0.325 to 0.591] for 90 mg). After the crossover at 90 days, participants initially assigned to placebo showed a moderate to large improvement compared to their prior trajectory (paired Student *t*-test *p* < 0.001, d = 0.66 [0.428 to 0.89]), their mood changing slightly more positively even compared to the group that started on 90 mg (Wilcoxon rank-sum test *p* = 0.053, δ = 0.17 [0 to 0.33]). The mixed-model semi-parametric approach in the SA population revealed a significant group-visit interaction term (*p* < 0.001) but no significance for the group term (*p* = 0.168) nor for the visit term (*p* = 0.232), highlighting the time-dependent nature of the effect. These results are illustrated in Figure 8.

**Figure 9:**
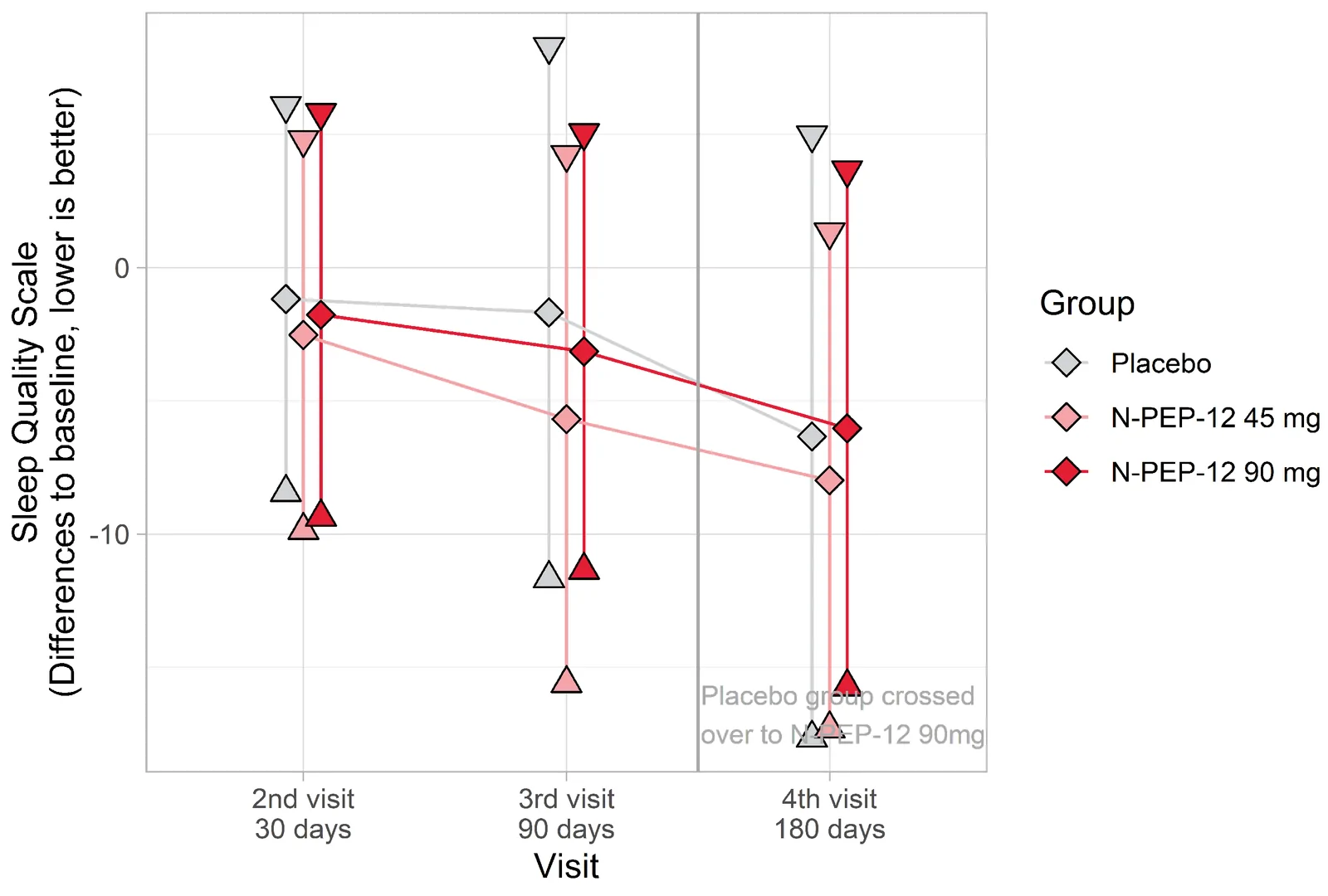
Distribution of Sleep Quality Scale scores. The diamond represents the mean, and the inward triangles represent the standard deviation referenced against the mean. Data from the Intention-To-Treat (ITT) population.

Because lower scores on the Sleep Quality Scale indicate better sleep, negative differences (including effect sizes) reflect improvement. The placebo group did not show a significant change up to the third visit (Wilcoxon signed-rank test, *p* = 0.38). In contrast, both the 45 mg (Wilcoxon rank-sum test, *p* < 0.001, δ = −0.296, 95% CI [−0.444 to −0.133]) and 90 mg (*p* = 0.034, δ = −0.185, 95% CI [−0.343 to −0.016]) N-PEP-12 groups demonstrated significantly greater improvements compared to placebo, with small to moderate effect sizes. Following crossover, participants initially assigned to placebo also showed a significant improvement (Wilcoxon signed-rank test, *p* = 0.013, *r*_rb_ = −0.309 [−0.515 to −0.068]), their improvement in sleep quality being comparable to the one observed by the 90 mg group during their first 90 days of treatment (Wilcoxon rank-sum test *p* = 0.158, δ = −0.123 [−0.287 to 0.047). The sensitivity analysis revealed a significant effect of the visit term (*p* = 0.048), without significant visit-group interaction (*p* = 0.358) or treatment term effect (*p* = 0.415). The corresponding distributions are shown in Figure 9.

**Figure 10:**
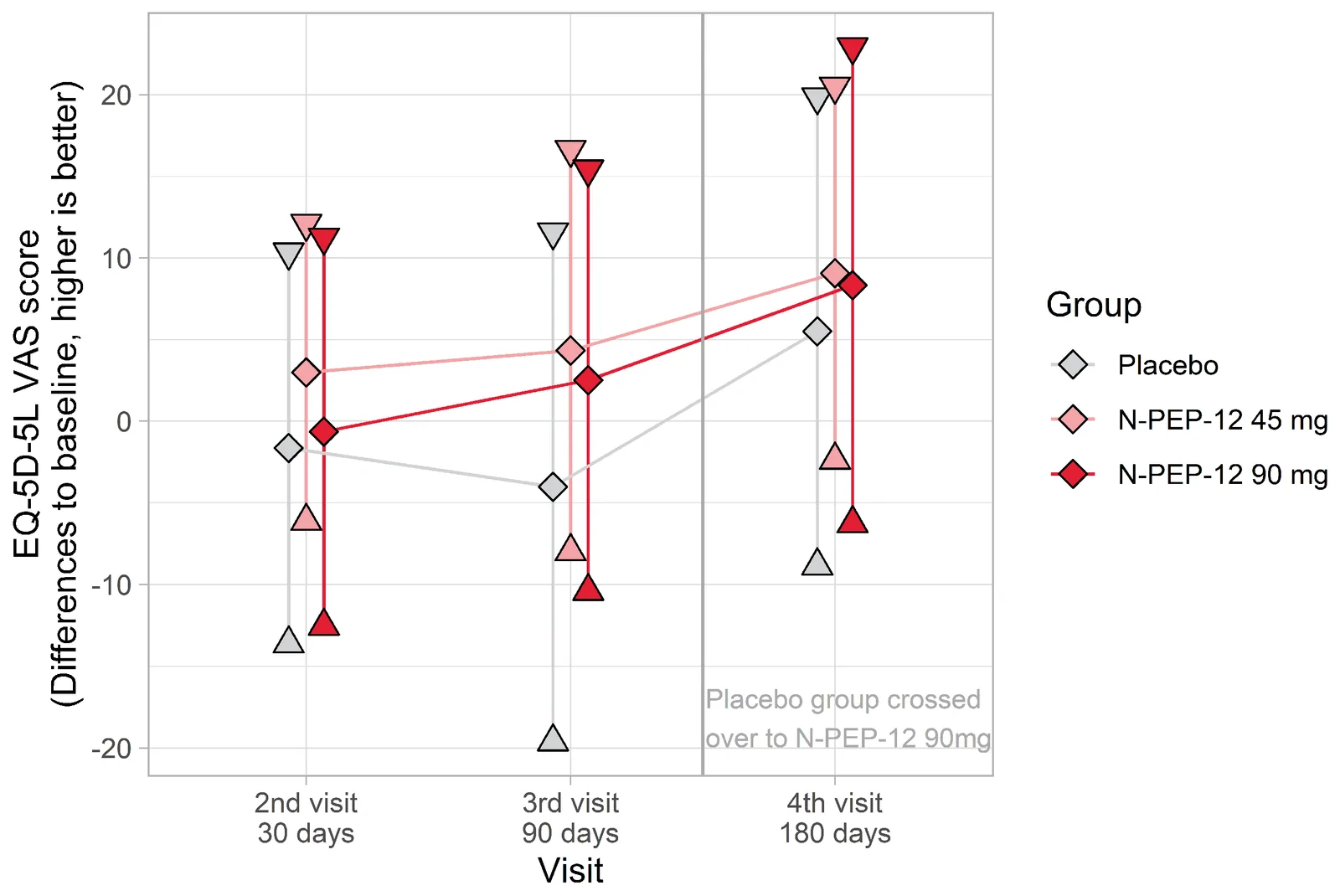
Distribution of EQ-5D-5L Visual Analog (VAS) values. The diamond represents the mean, and inward triangles represent the standard deviation referenced against the mean. Data from the Intention-To-Treat (ITT) population.

Lastly, we analyzed the Visual Analog Scale (VAS) from the EQ-5D-5L questionnaire, in which participants rated their current health on a scale from 0 (worst imaginable health) to 100 (best imaginable health). During the first 90 days, the placebo group showed a decrease in quality of life (paired Student’s *t*-test, *p* = 0.017, d = −0.257, 95% CI [−0.468 to −0.045]). In contrast, participants receiving N-PEP-12 at both 45 mg and 90 mg demonstrated significant improvements, both relative to baseline (*r*_rb_ = 0.491 for 45 mg and *r*_rb_ = 0.385 for 90 mg) and compared to the placebo group (Wilcoxon rank-sum test *p* < 0.001, δ = 0.372 and δ = 0.334, respectively). Following crossover, participants initially assigned to placebo showed a marked improvement compared to their prior trajectory (Wilcoxon signed-rank test, *p* < 0.001, *r*_rb_ = 0.625 [0.44 to 0.759]). This improvement was significantly greater than that observed in participants who received 90 mg N-PEP-12 from baseline (Wilcoxon rank-sum test, *p* = 0.001, δ = 0.283, 95% CI [0.119 to 0.432]). The mixed model applied over the sensitivity analysis population only revealed a tendency towards statistical significance for the interaction term (*p* = 0.054), with no significance for the visits (within-group) factor (*p* = 0.465) nor for the treatment arm (between-group) factor (*p* = 0.808). These results are illustrated in Figure 10.

### 3.3. Safety

A total of 20 adverse events (AEs) were reported between enrollment and the third visit, with a similar distribution across study groups (placebo: *n* = 7; N-PEP-12 45 mg: *n* = 8; N-PEP-12 90 mg: *n* = 5; χ^2^ test, *p* = 0.686). No adverse events were recorded in any group between the third and fourth visits, and no participant reported more than one AE.

Individual AEs, together with their severity, assessed relationship to study drug, outcome, and whether they led to withdrawal, are summarized in Supplementary Table S1, and their distribution by treatment group and study visit is shown in Supplementary Figure S1. All reported AEs were graded as mild or moderate in severity, no severe or serious adverse events occurred, and no participant died during the trial. None of the characterized AEs were assessed as related to the study drug. Eight participants discontinued the trial following an adverse event (placebo: *n* = 2; N-PEP-12 45 mg: *n* = 4; N-PEP-12 90 mg: *n* = 2); the remaining discontinuations were unrelated to adverse events (Figure 1).

## 4. Discussion

This randomized, placebo-controlled trial evaluated the effects of N-PEP-12 supplementation on attentional performance, cognitive function and mental wellbeing in healthy middle-aged and older adults with subjective cognitive complaints.

Our findings suggest that N-PEP-12 is associated with consistent improvements across multiple cognitive domains, particularly attentional performance and memory, as well as across broader aspects of mental wellbeing, including stress, mood, sleep quality and self-perceived health.

When it comes to cognitive aspects, the effects were observed in attentional performance, with participants receiving N-PEP-12 showing clear improvements compared to the placebo group. Similar results were noticed in working memory, where both Digit Span tasks (Forward and Backward) improved in the treatment groups. Together, these findings support the idea that N-PEP-12 may help maintain or enhance cognitive function even in individuals without clinically significant impairment. These findings are in line with the limited evidence available in older adults without diagnosed cognitive impairment, where similar improvements have been reported in individuals experiencing subjective cognitive complaints [14,36]. In addition, our results are comparable with previous studies conducted in clinical populations, such as stroke, for example, where N-PEP-12 has been shown to support cognitive recovery [16].

These findings were supported by a multivariate non-parametric repeated-measures model applied jointly across the three primary outcomes. This model demonstrated a significant group-by-visit interaction (*p* = 0.003) and a significant effect of visit (*p* < 0.001), without a significant main effect of treatment arm (*p* = 0.133), indicating that the benefit of N-PEP-12 is time-dependent and emerges over the course of treatment rather than representing a constant between-group difference. Notably, this result was obtained in a sensitivity analysis population in which missing data were imputed under the least favorable assumptions for the active arms, suggesting that the finding is robust to plausible departures from a missing-at-random mechanism.

An important aspect of this study is that the observed benefits extended beyond cognition, with better mood, improved sleep quality and higher overall quality of life in the treatment groups. Improvements in perceived stress were observed in the primary analyses but were not confirmed in the sensitivity analysis, and this outcome should therefore be interpreted with particular caution. These findings are highly relevant, as subjective cognitive complaints often affect mood and quality of daily life [37,38] and our group has previously reported a measurable relationship between mood and quantitative electroencephalographic markers in a related N-PEP-12 cohort [39]. These observed benefits suggest that the effects of N-PEP-12 are not limited to cognitive test performance but may extend to a more general sense of mental wellbeing. While evidence in humans remains limited, preclinical studies using derivatives of N-PEP-12 have also shown improvements in memory and stress response in animal models. These findings may help to explain the combined cognitive and wellbeing, more precisely stress-related improvements observed in our study [40].

Participants initially assigned to placebo showed improvement after being switched to active treatment at Day 90, reaching levels broadly comparable to those of participants who received N-PEP-12 from baseline. While this pattern is consistent with a treatment effect, it does not constitute confirmatory evidence. From Day 90 onward, the study had no concurrent placebo group and the observed within-participant changes could equally reflect period effects, continued practice on the test battery, regression to the mean, or altered expectations following a protocol-specified change in treatment. This phase should therefore be read as an uncontrolled extension that is descriptively consistent with the randomized findings, rather than as independent confirmation of them. The randomized, placebo-controlled comparison over the first 90 days remains the basis for the conclusions of this trial.

Interestingly, most of the improvements were seen in the first 90 days of treatment, after which these effects appeared to stabilize. This may indicate a plateau effect, with the initial benefits being maintained but not further enhanced by continued supplementation. Because no concurrent placebo group remained beyond Day 90, this observation is exploratory and hypothesis-generating only. However, these observations would need to be explored in future studies due to the lack of comparable data in this specific context.

Taken together, these findings contribute to a growing but still limited body of evidence on the potential role of N-PEP-12 in individuals with subjective cognitive complaints, particularly in the early stages, before clinically significant impairment develops.

### 4.1. Mechanistic Considerations and Pharmacokinetic Limitations

The mechanisms through which an orally administered peptide preparation might influence cognitive performance warrant explicit discussion, particularly because, to our knowledge, no human pharmacokinetic data for N-PEP-12 have been published and none were generated in the present trial. Therefore, we outline the plausible routes while being clear about the limits of the available evidence.

The first question is whether the peptide fraction survives gastrointestinal digestion. Dietary proteins and larger peptides are extensively hydrolyzed by gastric and pancreatic proteases and by brush-border peptidases, and the prevailing view is that intact absorption of larger oligopeptides in the healthy adult gut is limited [41]. Small di- and tripeptides, in contrast, are absorbed efficiently and in substantial quantity via the proton-coupled oligopeptide transporter PepT1 (SLC15A1), and a proportion escape cytosolic hydrolysis and reach the portal circulation intact [41]. Since N-PEP-12 comprises low-molecular-weight peptides rather than intact proteins, partial survival of the shorter peptide species is plausible. The formulation used here was not gastro-resistant, so the peptide load was exposed to the full gastric environment, and no direct demonstration of intact absorption exists for this preparation.

The second question is whether any absorbed component reaches the central nervous system. Passage across the blood–brain barrier is restrictive for hydrophilic peptides, and the evidence supporting central penetration of the parent preparation, Cerebrolysin, derives predominantly from parenteral administration in preclinical models and cannot be extrapolated to oral dosing in humans. Central effects need not, however, require intact peptides to reach the brain parenchyma. Free amino acids delivered by the preparation are absorbed by well-characterized transport systems and can serve as substrates for neurotransmitter synthesis and cerebral energy metabolism, and amino acid metabolic pathways are themselves altered in cognitive aging [19]. Some amino acid-derived compounds may additionally modulate cholinergic tone directly, as shown in vitro for creatine and taurine acting as competitive inhibitors of acetylcholinesterase [18]. Indirect signaling, including peripheral neuroendocrine and immune-mediated routes and gut-derived afferent signaling, offers a further plausible pathway that does not require blood–brain barrier passage. In preclinical work, an N-PEP-12 derivative improved cognitive performance and the acute stress response in aged rats subjected to chronic social isolation, a pattern consistent with the combined cognitive and stress-related improvements observed here [40].

The absorption, distribution, metabolism, and bioavailability of N-PEP-12 in humans remain uncharacterized. We are unable to state the systemic exposure achieved at either the 45 mg or 90 mg dose, the plasma half-life of any component, or whether the dose–response relationship suggested by our effect size estimates reflects differential systemic exposure. These data are not available, and their absence constrains mechanistic interpretation of our findings. Dedicated pharmacokinetic characterization of this preparation, including identification of the absorbed species, should be regarded as a prerequisite for a fuller mechanistic account.

Moreover, because N-PEP-12 is derived from mammalian neural tissue, its safety profile warrants comment beyond the adverse event counts reported in Section 3.3. The source material is purified porcine brain tissue. Porcine species are not affected by naturally occurring transmissible spongiform encephalopathy, and the manufacturer applies controlled sourcing and processing to the raw material. A further consideration is hypersensitivity, since peptides of xenogeneic origin can in principle act as haptens or antigens and rare anaphylactic reactions have been reported following intravenous administration of the parent preparation Cerebrolysin [42]. The oral route used in this trial substantially reduces that risk relative to parenteral administration and is consistent with the adverse event profile we observed: of the 20 adverse events recorded (Section 3.3), none were hypersensitivity reactions, and their distribution did not differ meaningfully between placebo and active arms. A trial of this size and duration is nonetheless not powered to exclude rare reactions, and this is noted in Section 4.2. 

### 4.2. Study Limitations

Several limitations should be considered. Firstly, some of the outcomes, such as stress, mood and sleep, were based on self-reported measures, which may be influenced by personal perception and the time of data collection. Also, participants were included based on self-reported subjective cognitive complaints, which may vary in severity and causes across individuals, therefore leading to a somewhat heterogeneous population in terms of baseline cognitive difficulties.

Another limitation is the narrow availability of comparable studies in healthy middle-aged and older adults with subjective cognitive complaints. While this highlights the novelty of our work, it also restricts direct comparisons with previous research and makes the interpretation of some observed effects more exploratory in nature.

Several further limitations should be noted. The detailed chemical composition, molecular-weight distribution, batch-specific analytical data, and quality-control specifications of N-PEP-12 are proprietary to the manufacturer and were not available to the investigators, which constrains the extent to which the preparation could be independently characterized or reproduced by other groups. No pharmacokinetic data were collected, so systemic exposure and the identity of the absorbed species remain unknown. The multivariate repeated-measures framework used for the primary analysis yields omnibus test statistics but does not produce model-derived treatment contrasts or confidence intervals, so the magnitude of the treatment effect cannot be quantified from that model directly. Finally, the Day 90 to Day 180 phase was an uncontrolled extension rather than a randomized comparison, and inferences drawn from it are exploratory.

## 5. Conclusions

N-PEP-12 supplementation was associated with improvements in attention, working memory and multiple aspects of mental wellbeing in healthy middle-aged and older adults with subjective cognitive complaints. These benefits emerged progressively over the randomized, placebo-controlled 90-day period, consistent with a time-dependent treatment effect and supported by a combined repeated-measures model that remained significant under conservative imputation of missing data; a comparable pattern was observed descriptively in participants who switched from placebo to active treatment during the subsequent uncontrolled extension. All these findings suggest that N-PEP-12 may be a promising option to support cognitive function and overall wellbeing in aging populations, particularly in individuals experiencing early or subjective cognitive changes before the onset of clinically significant impairment. Given the currently limited evidence in such populations, further research is needed to confirm these results in different groups and to better understand the long-term effects of N-PEP-12 supplementation.

## Figures and Tables

**Table 1:** Baseline demographic and lifestyle characteristics of the study population.

Variable	Placebo	N-PEP-12 45 mg	N-PEP-12 90 mg	*p*-Value (Statistical Test)
Randomized (*n*)	92	92	92	
Completed (PP)	88	85	87	
Females	67	66	66	*p* = 0.982 (Chi^2^ test **)
Males	25	26	26	
Age (years) Mean ± SD	61.185 ± 7.342	61.315 ± 7.606	61.663 ± 7.337	*p* = 0.891 (Kruskal–Wallis test)
Family history of dementia	15	13	14	*p* = 0.919 (Chi^2^ test)
No family history of dementia	77	79	78	
Education—Primary	0	1	0	*p* = 0.937 (Fisher’s exact test)
Education—Secondary	13	11	14	
Education—High school	29	26	29	
Education—Post-secondary	7	11	11	
Education—Short-term higher ed	2	2	1	
Education—Bachelor’s degree	35	29	27	
Education—Master’s degree	4	7	7	
Education—PhD or equivalent	2	5	3	
Physical activity *—Sedentary	32	26	29	*p* = 0.106 (Fisher’s exact test)
Physical activity—Moderate	47	51	39	
Physical activity—Increased	12	11	23	
Physical activity—High	1	4	1	
BMI (kg/m^2^)	26.87 ± 2.464	26.88 ± 2.267	27.239 ± 2.083	*p* = 0.59 (Kruskal–Wallis test)
Sleep hours ≥ 6 h/night	71	79	76	*p* = 0.302 (Chi^2^ test)
Sleep hours < 6 h/night	21	13	16	
Tobacco use	18	12	15	*p* = 0.488 (Chi^2^ test)
No tobacco	74	80	77	
Alcohol use	4	5	5	*p* > 0.999 (Fisher’s exact test)
No alcohol	88	87	87	
Other substances use	8	11	11	*p* = 0.729 (Chi^2^ test)
No use of other substances	82	81	79	
Not answering regarding other substances	2	0	2	

* Physical activity—Sedentary (<1/week); Moderate (1–2 days/week); Increased (5–6 days/week); High (daily). ** Chi-square tests were used where all expected cell counts were ≥5, and Fisher’s exact test (Freeman–Halton extension) otherwise, in 3 × 2 or 3 × k contingency tables. Participants who did not answer the item on other substance use (placebo *n* = 2; N-PEP-12 90 mg *n* = 2) were excluded from that comparison, which was performed on a 2 × 3 table.

**Table 2:** Baseline cognitive and patient-reported outcome measures.

Variable	Placebo (*n* = 92)	N-PEP-12 45 mg (*n* = 92)	N-PEP-12 90 mg (*n* = 92)	*p*-Value
TAP—Alertness	36.413 ± 8.146	36.239 ± 8.363	38.239 ± 9.492	*p* = 0.234
TAP—Attention	42.609 ± 10.516	40.707 ± 10.757	41.261 ± 9.634	*p* = 0.48
TAP—Memory	43.674 ± 14.948	41.935 ± 13.403	42.913 ± 13.56	*p* = 0.912
WAIS—Digit Span Forward	7.815 ± 2.399	7.489 ± 1.913	7.304 ± 1.982	*p* = 0.439
WAIS—Digit Span Backward	5.467 ± 1.901	5.435 ± 1.762	5.293 ± 1.831	*p* = 0.699
Perceived Stress Scale	17.315 ± 7.021	15.207 ± 5.729	16.207 ± 6.591	*p* = 0.185
BMIS Total score	11.565 ± 8.853	13.859 ± 7.496	11.815 ± 8.343	*p* = 0.149
Sleep Quality Scale	17.837 ± 13.23	14.467 ± 11.876	12.826 ± 9.856	*p* = 0.052
EQ-5D-5L VAS score	78.467 ± 15.641	80.304 ± 11.877	81.043 ± 12.57	*p* = 0.71

Data are presented as Mean ± Standard Deviation. Statistical significance was evaluated across all groups using the Kruskal–Wallis test. Abbreviations: BMIS, Brief Mood Introspection Scale; EQ-5D-5L VAS, EuroQol 5-Dimension 5-Level Visual Analog Scale; TAP, Test for Attentional Performance—median reaction time with no signal (TAP—Alertness), omission T-scores (TAP—Attention) and memory omission T-scores (TAP—Memory); WAIS, Wechsler Adult Intelligence Scale.

## Data Availability

The study protocol is publicly available via the ISRCTN registry (ISRCTN10514154). De-identified participant data and analysis code will be made available by the corresponding author upon reasonable request.

## Supplementary Materials

The following supporting information can be downloaded at: https://www.mdpi.com/article/10.3390/nu18162739/s1, Excel file—supplementary outcomes. Table S1: Adverse events by treatment group, Day 0–180, with severity, relationship to study drug, outcome and withdrawal status for each event; Figure S1: Number of participants with adverse events by treatment group and study visit. Table S2: CONSORT 2025.
